# Ultrasonic Film Rehydration Synthesis of Mixed Polylactide Micelles for Enzyme-Resistant Drug Delivery Nanovehicles

**DOI:** 10.3390/polym14194013

**Published:** 2022-09-25

**Authors:** Darya A. Stepanova, Vladislava A. Pigareva, Anna K. Berkovich, Anastasia V. Bolshakova, Vasiliy V. Spiridonov, Irina D. Grozdova, Andrey V. Sybachin

**Affiliations:** 1 Leninskie Gory, 1-3, Chemistry Department, Lomonosov Moscow State University, 119991 Moscow, Russia; 2 Frumkin Institute of Physical Chemistry and Electrochemistry, Russian Academy of Sciences, 119071 Moscow, Russia

**Keywords:** polylactide micelles, synthesis, self-assembly, ultrasonication, drug delivery, paclitaxel, polylactide, cytotoxicity, Paclitaxel-Teva, taxol

## Abstract

A facile technique for the preparation of mixed polylactide micelles from amorphous poly-D,L-lactide-block-polyethyleneglycol and crystalline amino-terminated poly-L-lactide is described. In comparison to the classical routine solvent substitution method, the ultrasonication assisted formation of polymer micelles allows shortening of the preparation time from several days to 15–20 min. The structure and morphology of mixed micelles were analyzed with the assistance of electron microscopy, dynamic and static light scattering and differential scanning calorimetery. The resulting polymer micelles have a hydrodynamic radius of about 150 nm and a narrow size distribution. The average molecular weight of micelles was found to be 2.1 × 10^7^ and the aggregation number was calculated to be 6000. The obtained biocompatible particles were shown to possess low cytotoxicity, high colloid stability and high stability towards enzymatic hydrolysis. The possible application of mixed polylactide micelles as drug delivery vehicles was studied for the antitumor hydrophobic drug paclitaxel. The lethal concentration (LC50) of paclitaxel encapsulated in polylactide micelles was found to be 42 ± 4 µg/mL—a value equal to the LC50 of paclitaxel in the commercial drug Paclitaxel-Teva.

## 1. Introduction

The continuous search for effective vehicles for drug delivery is one of the key tasks of modern medicine [1,2,3]. These vehicles should satisfy the following requirements: biocompatibility, biodegradation, long-term circulation in biological media, high capacity of drug and possibility for the delivery of complex multicomponent (including inmixelanous) drugs [4,5,6,7]. In addition, these carriers should be capable of target delivery either by “active” routes or by “passive” targeting [8,9]. The former route requires chemical modification of the carrier with vector molecules that could interact with target sites on target cells (e.g., antigen–antibody coupling) [10]. Therefore, the developed nanocarriers should have functional groups, for example, amino groups, that are able to be easily modified to impart the particles’ vector properties [11].

The latter way is based on size of the nanocarrier that should ensure the accumulation of the drug in areas of inflammation with enlarged vascular pores and prevent the accumulation in normal tissue. Thus, the optimal size for the nanocontainer was reported to be in the sizes interval 100–400 nm [12,13,14].

A large number of bioactive substances are hydrophobic, so their encapsulation requires the existence of sufficient hydrophobic areas in nanocontainers or these bioactive molecules could be attached to hydrophilic carriers by chemical bonds [15,16,17]. Among nanovehicles—cyclodextrins, conventional surfactant-based micelles, liposomes, polymer micelles—only the last two classes may be suggested to be used as carriers with both “active” and “passive” targeting. 

Cyclodextrins are cyclic oligosaccharides that are able to encapsulate hydrophobic molecules into their hydrophobic interior [18]. This interior is determined by the number of glucose subunits in cyclodextrins but the total volume for the incorporation of hydrophobic substances is very small [19]. Hydroxyl groups of cyclodextrins could be functionalized to accumulate several molecules of oligosaccharides in aggregate and increase the total number of bioactive substances in this aggregate particle [20].

Cationic, anionic and non-ionic surfactants and their mixtures are able to form micelles if their concentration in solution exceeds the critical micelle concentration (CMC) [21]. Hydrophobic tails form a core suitable for encapsulation of hydrophobic molecules. Relatively high CMC values for traditional surfactants result in decomposition of micelles on individual molecules due to dilution, so their application for drug delivery is very restricted. In general, conventional surfactants are used to stabilize nano- and microemulsions for drug delivery [22,23,24].

Liposomes are spherical lipid bilayer vesicles [25]. They are able to encapsulate hydrophobic molecules in areas of fatty tails, manipulation with lipid membrane composition allows one to modify their surface with vector molecules, to impart to liposomes stimulus-responsiveness and the size of vesicles could be varied in a wide range from tens to hundreds of nanometers [25,26,27,28,29,30,31]. However, liposomes have poor colloid stability and low encapsulation efficiency of hydrophobic substances. Partially, these problems could be solved by modification of liposomes with polymers such as polyethyleneglycol (PEG) or accumulation of dozens of liposomes on polymer carriers [32,33,34,35]. 

Polymer micelles are formed from copolymers with hydrophilic and hydrophobic blocks [17,36,37,38]. Self-assembled structures of these macromolecules are governed by the length of blocks, their ratio and the chemical nature of the polymer chain [39]. The micelles formed by block copolymers usually have a hydrophobic core stabilized by a hydrophilic shell. Polymer micelles could be spheres, disks, cylinders, etc. [40,41]. The micelle core serves as a depot for the solubilization of hydrophobic drugs, and the hydrophilic shell protects against the effects of the immune system [42,43]. In contrast to surfactant-based micelles, the CMC of polymer micelles is several magnitudes lower, and some polymer micelles with “frozen” structures do not decompose at all after dilution [44,45]. Polymer micelles are prospective nanocarriers for drug delivery with prolonged circulation in the human body—the hydrophilic part of the block copolymer is responsible for the “masking” of micelles from the reticuloendothelial system and preventing the adsorption of proteins [46,47]. Polymeric micelles are able to avoid capillary filtration [48] and kidney filtration [49]. The most common hydrophilic block of an amphiphilic copolymer for making micelles is PEG. It is approved for parenteral use by the FDA and is widely used in a variety of biomedical and nutritional applications [49,50]. The key advantage of PEG is its low toxicity [49,51].

The hydrophobicity, biodegradability and availability of polylactide make it promising as a drug carrier. PLA has been extensively studied for its loading capacity and release of hydrophobic drugs. In the course of block copolymerization with hydrophilic polyethylene glycol, an amphiphilic polymer is obtained, capable of self-organizing in an aqueous solution into micelles with a polylactide hydrophobic core and a PEG shell.

Lactide–ethylene glycol block copolymer micelles are widely used to solubilize paclitaxel, a hydrophobic anticancer drug. In this case, it is possible to significantly (> 5000 times) increase the solubility of the drug in comparison with the traditional dosage form [47]. Another paper describes the preparation of PLA-PEG micelles with aldehyde groups at the ends of PEG chains to introduce a surface charge and amino acid residues into the hydrophilic shell [52]. In this way, vector properties can be imparted to micelles. The introduction of glucose molecules into the shell of micelles for targeted delivery through glycoreceptors was also studied [53]. It was also shown that at a temperature of 37 °C in an isotonic solution of PLA-PEG micelles retains their colloidal stability [54]. All the described properties of lactide–ethylene glycol block copolymer micelles make them potentially useful for targeted drug delivery.

It is known that the efficiency of drug delivery systems is significantly affected by their physicochemical characteristics, such as particle size and surface charge. They affect the adhesion of nanocontainers and their interaction with cells [55].

Early works devoted to the study of liposomal nanocontainers noted the tendency of positively charged particles to accumulate and stay in the tumor tissue longer than neutral or negatively charged ones [56,57,58].

In this work, we focused on preparation of polymer micelles with positively charged groups on their PLA core. For this purpose, the micelles were prepared from a mixture of PLA-PEG copolymer and amino-terminated polylactide, and as a result polylactide micelles with amino groups (PLAMs) were obtained and thoroughly investigated. There are several approaches to form polymer micelles.

The routine technique for the synthesis of PLAMs includes dissolving the polymers in tetrahydrofuran (THF) and further additions of portions of water with a final step of dialysis for the elimination of the organic solvent [59,60]. This procedure takes several days and results in low concentrations of PLAMs and high polydispersity of the micelles. Moreover, the final sample may contain traces of organic solvents. The successful attempts to obtain narrowly distributed mixed micelles with relatively small size were successful either by use of oligomers of polylactide or by exclusion of polydisperse large fractions by nanofiltration [61]. There exists a direct dissolution approach and, if the copolymer is relatively water soluble, this approach can be used to form micelles. In this case, the copolymer is simply added to the aqueous medium at a concentration above the CMC [62]. However, it should be taken into account that for amphiphilic block copolymers (including PLA-PEG), CMC is in the range of 10^−6^–10^−7^ M. Due to the extremely low solubility of PLA in water, this approach is not applicable. In addition, this approach does not allow one to control the particle size and homogeneity.

Here, we demonstrate a novel approach to the synthesis of PLAMs via an ultrasonication-mediated film rehydration technique and discuss the physicochemical characteristics of synthesized micelles and their potential to be used in the modern drug delivery field.

## 2. Materials and Methods

### 2.1. Reagents

**Figure 1:**
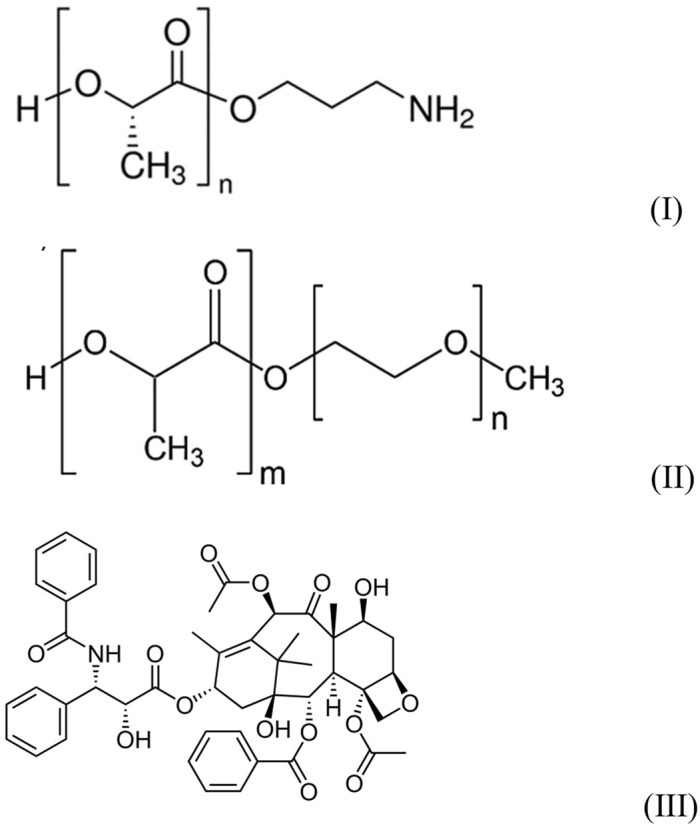
Structures of reagents.

Amino-terminated poly-L-lactide (PLA-NH_2_) with Mn = 2500  and PDI ≤ 1.3 (see Figure 1I), polyethyleneglycol methyl ether-block-poly-D,L-lactide (PLA–PEG) with polyethylene glycol Mn = 2000 and polylactide Mn = 2000  and PDI < 1.4 (Figure 1II), from Sigma-Aldrich (St. Louis, MO, USA), were used as received. 

The antibiotic paclitaxel (Figure 1III) from ApexBio (Houston, TX, USA) and the pharmaceutical form of paclitaxel (Taxol^®^)- Paclitaxel-Teva (macrogol glyceryl ricinyl oleate, anhydrous citric acid and absolute ethanol containing composition), from Pharmachemie (Haarlem, The Netherlands) were used as received.

The enzyme trypsin, sodium polystyrenesulfonate (PSS) with Mw = 200,000, sodium acetate and tris-hydroxyaminomethan (Tris) from Sigma-Aldrich (St. Louis, MO, USA) were used as received.

Bidistilled water (DI water) was used in all experiments. 

### 2.2. Standard Procedure of Preparation of PLAMs

PLAMs with PLA-NH_2_/PLA-PEG molar ratio of 3:7 and concentration of 0.047 mg/mL were prepared according to the solvent substitution technique described earlier [63]. Briefly, 20 mg/mL solutions of PLA-NH_2_ and PLA–PEO in THF were prepared. Then, a 1.5 mL mixture of these solutions was prepared with a final PLA-NH_2_ to PLA–PEO molar ratio of 3:7 and total molar concentration of polymers was 5 × 10^−5^ M. Finally, a portion of DI water was added to the thus-prepared mixture with intensive intermixing, so that the total content of water was 10 wt%. After 24 h of incubation under continuous intermixing, an additional portion of DI water was added with intensive intermixing, thus increasing the water content up to 20 wt%. After 24 h of incubation under continuous intermixing, the polylactide mixture was dialyzed against DI water for 1 week with a daily change of external solvent. As a result, 3.4 mL of PLAM water suspension with polylactide concentration of 2.2 × 10^−5^ M or 0.047 mg/mL was prepared (PLAMs–cl).

### 2.3. Polylactide Film Rehydration Method for PLAM Preparation

First, 9.25 mg of PLA-NH_2_ and 34.6 mg of PLA-PEG were weighed and placed in a round bottom flask. Then, 3 mL of the THF was added to the flask to dissolve the polylactides. Then, the organic solvent was evaporated at 37 °C under vacuum using a Laborota 4000 rotor evaporator (Heidolph, Schwabach, Germany). A thin film of mixed polylactides was formed on the bottom of the flask. Three identical batches were prepared and then were subjected to different external actions. The formed film of the first batch (PLAMs–v) was rehydrated with 2.19 mL of deionized bidistilled water and was vigorously shaken on a Genie 2 vortex (Scientific Industries, Bohemia, NY, USA) for 10 min so that the polylactide film was completely transferred to the suspension fraction. The film of the second batch (PLAMs–sb) was also rehydrated with 2.19 mL of DI water and homogenized in a Sapphire ultrasonic bath (Sapphire, Russia) with power 550 W and frequency 35 kHz in one cycle for 10 min. Finally, the third batch (PLAMs–us) was rehydrated with 2.19 mL of DI water and homogenized in a cylindrical cell with a titan tip CP-750 ultrasonic dispergator (Cole-Parmer, Vernon Hills, IL, USA) with frequency 20 kHz and amplitude value 20% for 10 min. As a result, three suspensions of PLAMs with PLA-NH_2_/PLA-PEG molar ratio 3:7 with concentration 20 mg/mL were prepared.

PTX-loaded micelles (PLAMs–PTX) were synthesized according to the tip sonication procedure described above. The calculated amount of PTX in THF was added to the mixture of the polylactides in THF prior to evaporation of the organic solvent. As a result, the suspension of PLAMs–PTX with 5% mass fraction of PTX was prepared.

### 2.4. Methods

The hydrodynamic radii were obtained by dynamic light-scattering using ZetaPlus (Brookhaven, Holtsville, NY, USA) equipment and DynaLS software package for size distribution analysis (contin method).

The diffusion coefficients for the PLAMs were obtained by dynamic light-scattering measurements using a complex laser light goniometer (Photocor Instruments, Moscow, Russia) equipped with a He−Ne laser and data processing was performed using DynaLS software version 2.7.1 [64].

Molecular weight of micelles was measured by static light-scattering (SLS) using a goniometer with a He–Ne laser (λ = 633 nm) and photomultiplier supplied with Photocore software.

The electrophoretic mobility of particles (EPM) was controlled by Brookhaven ZetaPlus (Brookhaven, Holtsville, NY, USA) equipment with software provided by the manufacturer.

IR spectra of samples were obtained using a Specord M-80 IR (Carl Zeiss, Jena, Germany). A 20 μL droplet of 1 mg/mL PLAMs suspension was deposited between two KBr glasses and the spectrum was recorded in absorption regime in a wavelength range from 400 to 2000 cm^−1^.

Microelectronic images of PLAMs were obtained with a Leo 912AB TEM with Omega filter (Carl Zeiss, Jena, Germany), 100 kV. The samples were prepared by deposition of a drop of the suspension on a TEM copper grid. The measurements were performed after drying of the sample.

Differential scanning calorimetry measurements were performed using DSC 204 F1 (Netzsch, Selb, Germany) at heating rate 10 °C/min, in a temperature range from 0 to 200 °C in inert atmosphere with argon flow rate 100 mL/min. The samples of PLAMs were lyophilized prior to measurements and 2 mg of samples were used. For the PLA-NH_2_ and PLA-PEG samples, no additional manipulations prior to DSC measurements were made.

UV–Vis spectroscopy of the samples was performed using a UV-mini 1240 spectrometer (Shimadzu, Kyoto, Japan) in wavelength range from 200 to 500 nm.

A cytotoxicity MTT test was carried on NCI/ADR-RES (formerly designated as MCF-7/ADR) cell culture. Cells were seeded in 96-well plates, 4000 cells per well, and were incubated with the substrate for 1 h in serum-free culture medium, each concentration in triplicate. Control wells contained no substrate. Then, the medium was replaced with a fresh portion supplemented with 10 wt% fetal bovine serum and the cells were cultured for an additional 3 days. The relative number of surviving cells was detected using a 3-(4,5-dimethylthiazol-2-yl)-2,5-diphenyltetrazolium bromide (MTT) assay [65]. The number of surviving cells was estimated as proportional to formazan concentration. The detection of formazan was performed with an Ultrospec 1100 spectrophotometer (Amersham Bioscience, Buckinghamshire, UK) by measuring the absorbance of the samples at 550 nm. Three micelle-free samples of cells were used as a control. Viability of control cells was taken as 100%. The fraction of surviving cells was calculated as the ratio of absorbance of the sample to the average absorbance of the control. Incubation time of suspensions with cells was 1.5 h.

The resistance of micelles to enzymatic hydrolysis was studied. The study of susceptibility to enzymatic hydrolysis was carried by the control of the hydrodynamic sizes of particles for 56 h in three temperature regimes (7 °C, 25 °C and 37 °C). 

The suspensions of bare PLAMs and PLAMs–PTX were prepared with a concentration of 0.5 mg/mL in a Tris buffer solution (c = 10^−2^ M) with pH 7. The hydrolyzing enzyme solution was added to each sample so that the concentration was 1 mg/mL.

The stability of PLAMs in media with different pH values in a range from 5 to 9 was controlled by measuring hydrodynamic sizes of particles for 1 week.

The control of release of PTX from PLAMs–PTX in buffer was carried out by the following procedure. The PLAMs–PTX were incubated in PBS buffer that was used for incubation with cells for 5 min and 1.5 h. The micelles were separated from the suspension using centrifugation tubes with filters with 100 K cut-off (Millipore, Burlington, MA, USA). The separated solution was analyzed using UV spectrophotometry.

## 3. Results

### 3.1. Optimization of the Sonication Procedure

**Figure 2:**
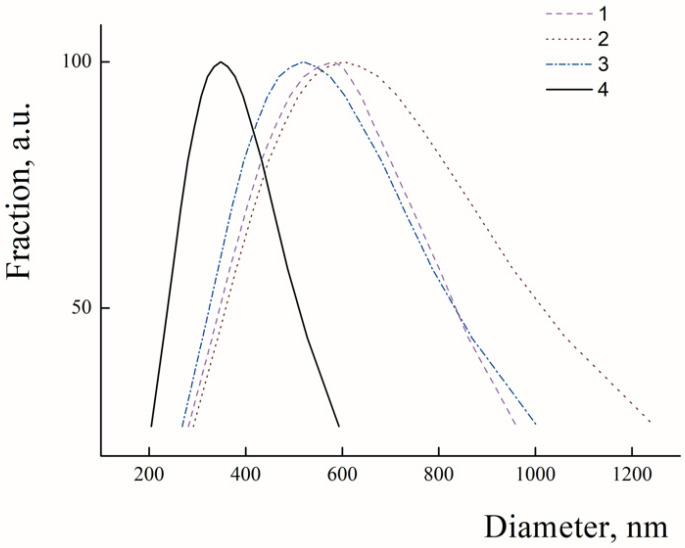
Size distributions in suspensions of PLAMs–cl (1); PLAMs–v (2); PLAMs–sb (3); and PLAMs–us (4). Concentration of PLAMs 0.5 mg/mL, Tris buffer with pH 7, 25 °C.

In the first step, average hydrodynamic diameters of the freshly prepared PLAMs were analyzed with use of dynamic light-scattering. In Figure 2, the PLAMs’ size distributions by intensity are presented. The mean hydrodynamic diameter of PLAMs–cl was found to be 582 nm with PDI 0.187 (see Figure 2). The sizes of aggregates of micelles detected during measurements were eliminated by software. This relatively high value and high polydispersity and presence of aggregates make application of the micelles prepared by this procedure in biomedicine quite doubtful. Further, this sample was used for reference measurements. The PLAMs–v sample was found to have hydrodynamic diameter of 603 nm with PDI 0.246. The enlarged size and polydispersity of micelles could be attributed to insufficient forces of film dispergation by vortexing to obtain particles with uniform distribution. For the PLAMs–sb samples, the mean diameter was found to be 513 nm with PDI 0.160. Thus, sonication allowed us to obtain a more narrow distribution of micelles’ sizes. However, the power of sonication was not enough to produce particles with smaller sizes. Finally, the mean diameter for the PLAMs–us was found to be 340 nm with PDI 0.110. For the nanocarriers of bioactive substances in drug delivery that are developed for a passive targeting delivery mechanism, one of the requirements is a size that should not exceed 400 nm [12]. Only one sample of PLAMs obtained via tip sonication satisfied this requirement. 

**Figure 3:**
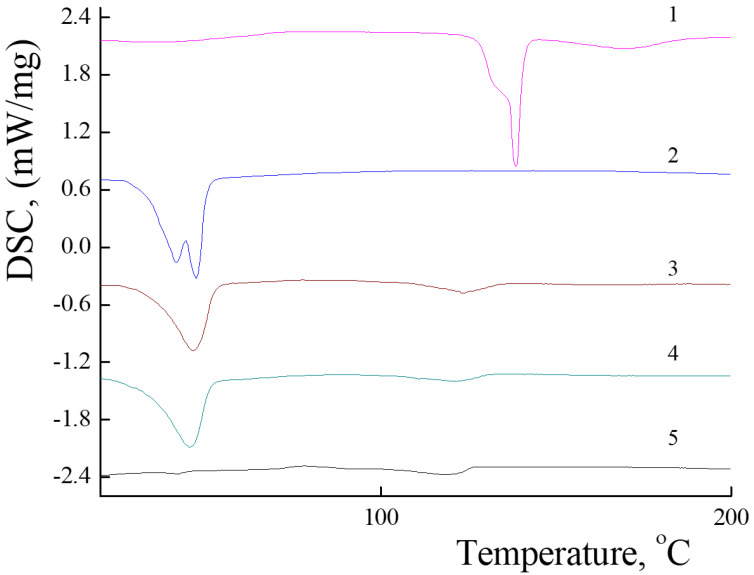
DSC curves for PLA-NH_2_ (1); PLA-PEG (2); PLAMs–v (3); PLAMs–sb (4); and PLAMs–us (5). Heating rate 10 °C/min.

The uniform distribution of the PLA-PEG and PLA-NH_2_ molecules is one of the key requirements determining their physicochemical properties and possible applications. Mixed PLAMs are formed from crystalline PLA-NH_2_ and amorphous PLA-PEG, so DSC was applied to analyze the distribution of polylactides in micelles’ cores. The DSC curve of PLA-NH_2_ (see Figure 3 curve 1) contains a melting peak at 138 °C with crystallization energy −48 J/g (for the details, see Figure S1 in the Supplementary Materials). These data correspond to the melting temperature (Tm) of poly-L-lactide. For the PLA-PEG sample (see Figure 3 curve 2), the endo-peak at 44 °C could be reflect the glass transition temperature (Tg) of poly-D,L-lactide or melting peak of PEG. The appearance of the broad peak could reflect reorganization of polylactide chains from a tense structure that could be formed due to transport conditions at temperatures above room temperature and fast cooling in the fridge to a structure with minimal free energy. The PLAMs–v and PLAMs–sb samples (Figure 3, curves 3 and 4, respectively) were also found to have no peak on DSC curves reflecting the melting of PLA-NH_2_, so it could be stated that crystalline polylactide was finely distributed in PLAMs and no crystalline phase was formed. However, both curves have endo-peaks at Tg of poly-D,L-lactide. So, it could be concluded that the distribution of polylactide chains in cores of these PLAMs results in formation of non-equilibrated stressed structures with high free energy.

For the sample of PLAMs–us, no peaks reflecting Tm of poly-L-lactide or Tg of poly-D,L-lactide were observed on the DSC curve (Figure 3, curve 5). Thus, we may conclude that PLA-NH_2_ and PLA-PEG macromolecules are uniformly distributed in the PLA core of PLAMs–us. In the control experiment for the PLAMs–cl sample, no endo-peaks on the DSC curve were found either (see Figure S2 in the Supplementary Materials).

DSC curves of PLAMs obtained by the solvent substitution method and tip sonication procedure have no peaks at Tg of poly-D,L-lactide and Tm of poly-L-lactide. So, these techniques allow one to obtain the equilibrium structures of PLAMs with uniformly distributed polylactide chains.

In comparison to gentle preparation techniques of PLAMs–cl and PLAMs–v without additional energy impact, the ultrasonic treatment could result in formation of peroxide groups or induce oxidation of the components in PLA-NH_2_/PLA-PEG mixtures [66,67]. To control the possible change in chemical composition of micelles, the IR spectrum of PLAMs–us was registered (see Figure S3 in the Supplementary Materials).

The peaks corresponding to vibrations of -C-O-C- bonds at 920–800 cm^−1^ and -C(O)-O-C- at 1690–1550 cm^−1^ reflecting PLA and PEG units were found on the spectrum. No peaks corresponding to vibration of –O-O- at 890 cm^−1^, alkyl peroxides at -C-O-O at 1030–1150 cm^−1^, primary hydroperoxides at 1488 and 1435 cm^−1^ and secondary peroxide at 1352–1334 cm^−1^ were observed. Thus, the tip sonication did not result in formation of reasonable detectable amounts of peroxide groups. 

Further, we will discuss only PLAMs–us as potential nanovehicles for drug delivery.

### 3.2. Characterization of PLAMs–us

**Figure 4:**
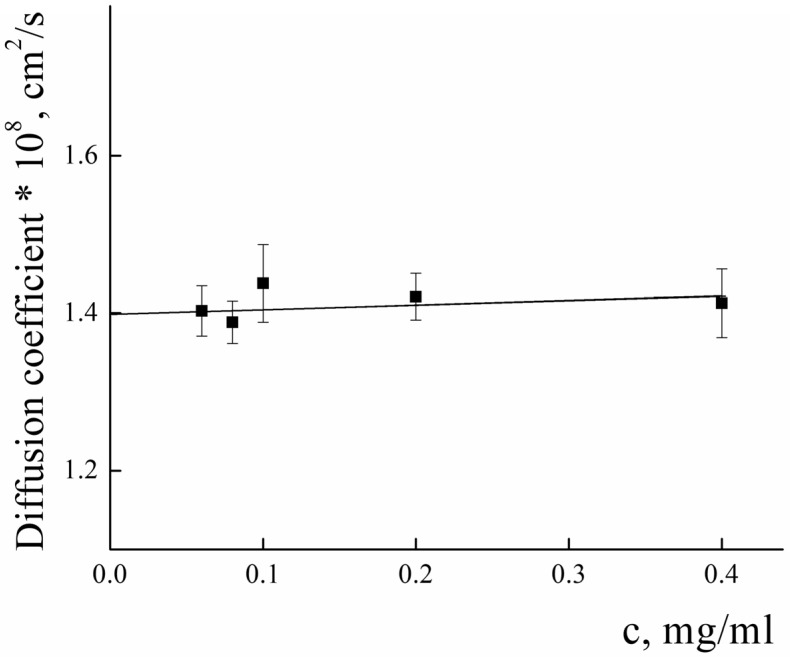
Concentration dependence of the diffusion coefficient of PLAMs–us in Tris buffer with pH 7, c = 0.01 M, 25 °C.

To analyze the structure of PLAMs–us, dynamic and static light-scattering measurements were performed. The concentration dependence of the diffusion coefficients (*D*) of PLAMs–us was measured in 0.01 M Tris buffer (pH = 7) with 0.15 M of NaCl solution. The results are presented in Figure 4. Extrapolation of the concentration dependence of *D* to zero concentration allowed us to estimate the resulting *D_0_* value (1.39 ± 0.01) × 10^−8^ cm^2^/s, which was used in calculations using the Stokes−Einstein equation:

(1) $$D=\frac{k_{B}T}{6\pi \eta R_{h}}$$

The calculated value of the hydrodynamic radius of PLAMs–us was found to be 170 ± 15 nm.

**Figure 5:**
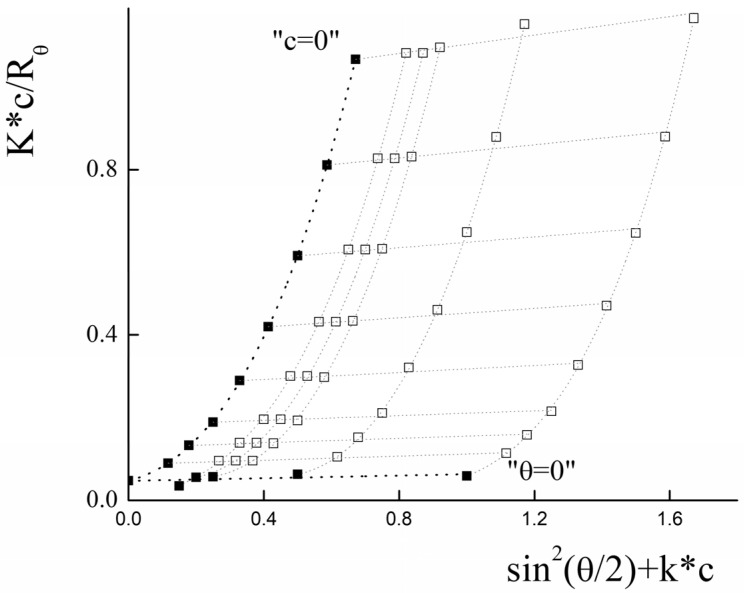
Zimm plot for PLAMs–us (blank squares correspond to experimental data, solid squares correspond to extrapolation data). Tris buffer with pH 7, c = 0.01 M, 25 °C.

Static light-scattering was used to determine the radius of gyration (*R_g_*) and molecular weight (*M_w_*) of PLAMs–us. The Zimm plot for micelles is presented in Figure 5.

For the large particles with a size bigger than *λ_0_*/20 (where *λ_0_* is instrumental laser wavelength), the intensity of light-scattering depends on both concentration of the sample and the scattering angle (*θ*). In this case, for the estimation of average molecular weight, the Debye equation should be used [68]:

(2) $$P^{-1}\left(\theta \right)=1+\frac{1}{3}\cdot <R_{g}{>}^{2}{\left(\frac{4\pi n_{0}}{\lambda_{0}}\right)}^{2}\cdot \sin^{2}\left(\frac{\theta }{2}\right)$$

where *P*(*θ*) is form factor, *n_0_* is refractive index of solvent. However, the analysis of the data using this equation was not successful. For the particles with molecular weight more than 10^6^ g/mol, the following general form of the Debye equation should be used [69]:

(3) $$P^{-1}\left(\theta \right)=1+\alpha_{1}\mu^{2}-\alpha_{2}\mu^{4}+\ldots$$

where *μ* $=\frac{4\pi n_{0}}{\lambda_{0}}\sin\left(\frac{\theta }{2}\right)$, $3\alpha_{1}=<R_{g}{>}^{2}$

Using the least squares method, it was found that optimal calculations could be achieved using polynomial of the third degree by $\mu^{2}$.

The experimental data and the results of the extrapolation are presented in Figure 5. The results of extrapolation gave us the following values: *R_g_* = (130 ± 10) nm and *M_w_* = (2.1 ± 0.1)∙10^7^ g/mol.

Using the SLS data, the aggregation number (*AN*) for PLAMs–us was calculated as:

(4) $$AN=\frac{M_{w}}{0.7\cdot M_{PLA-PEG}+0.3\cdot M_{PLA-NH2}}=6000\pm 300$$

The ratio of *R_g_* and *R_h_* corresponds to a form factor (ρ) that could be used to analyze the morphology of the particles. For PLAMs–us, ρ was calculated to be 0.76 ± 0.12, so the micelles could be considered as core–shell particles for which the ρ = 0.775 [70].

**Figure 6:**
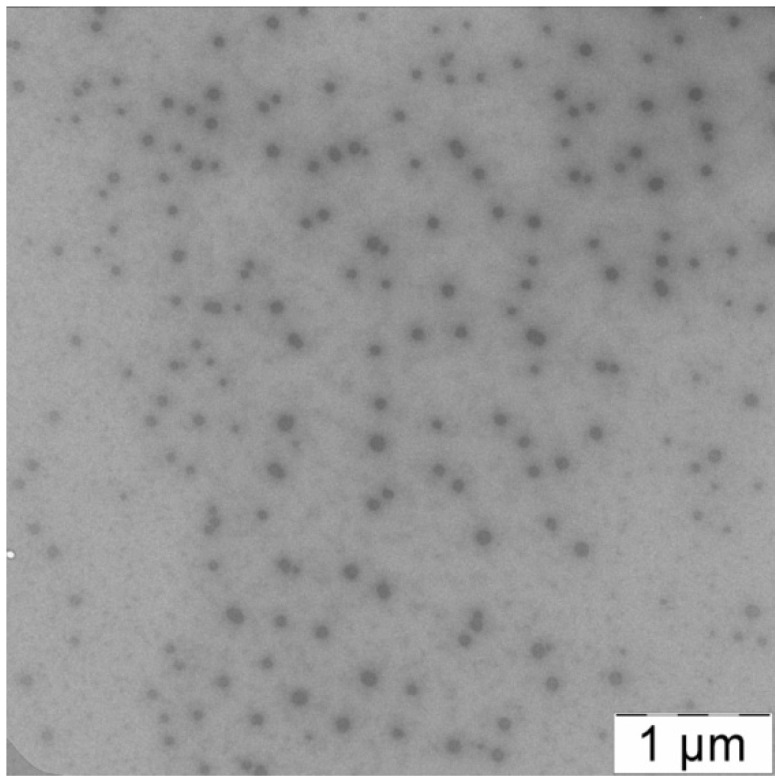
TEM image of PLAMs–us.

Additional information about morphology of PLAMs–us could be obtained via TEM imaging. The TEM image of PLAMs–us is presented in Figure 6. The micelles have an almost uniform shape with dense polylactide core and PEG corona. The average diameter of dark disks reflecting the micelle core was found to be 70 ± 15 nm. As TEM images reflect the top view of the sample, the disks could be attributed to the spherical shape of the micelle core that is in good agreement with the form factor obtained by a combination of static and dynamic light-scattering.

**Figure 7:**
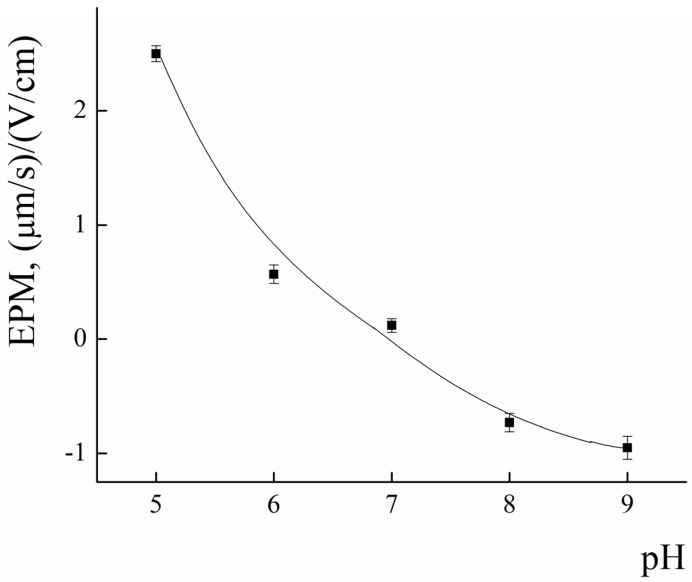
The pH dependence of EPM for PLAMs–us; 25 °C.

Incorporation of PLA-NH_2_ into polylactide micelles was performed to impart additional affinity of the PLAMs to negatively charged cell membranes and for further possible modification of the micelles by functionalization of NH_2_ groups. The amino groups of PLAMs are screened by PEG corona, so it is important to estimate the number of available amino groups. The EPM values of PLAMs–us were studied using laser microelectrophoresis to estimate surface charge of micelles. The dependence of the EPM of PLAMs–us upon the pH of the system is presented in Figure 7.

In acidic media at pH = 5, the EPM of PLAMs was found to be 2.5 ± 0.1 (μm/s)/(V/cm). With the increase in the pH, the values of EPM of PLAMs–us decreased and reached zero at pH = 7. A further increase in the pH results in slight recharging of the micelles up to the value −0.7 ± 0.1 (μm/s)/(V/cm) at pH 9. 

The explanation for such unusual behavior of the surface charge of PLAMs formed from cationic PLA-NH_2_ and neutral PLA-PEG could be as follows. An increase in the EPM in acidic media is attributed to protonation of the primary NH_2_ group. With the increase in pH, the share of protonated amino groups rapidly decreases. Negative values of the EPM could arise due to the traces of initiator of polylactide synthesis. A slight negative surface charge was reported for individual PLA-PEG micelles earlier [64].

**Figure 8:**
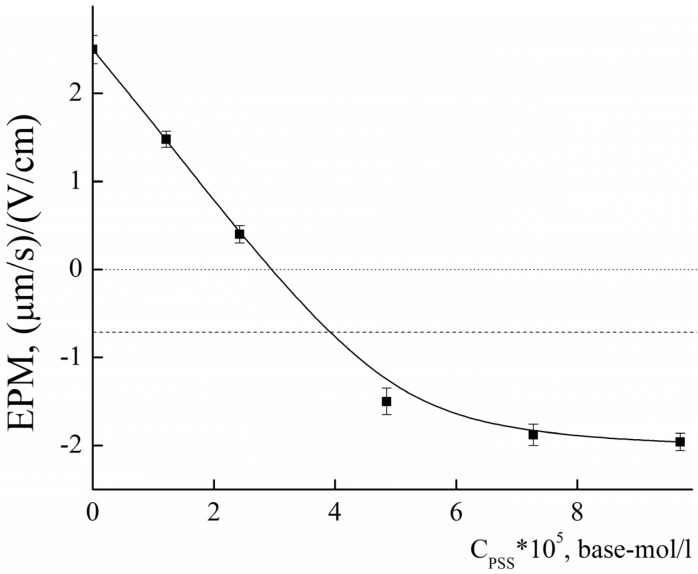
The dependence of EPM of PLAMs–us upon the concentration of added PSS; acetate buffer with pH 5, c = 0.01 M, 25 °C.

To assess the chemical availability of amino groups, the obtained PLAMs–us were titrated with an oppositely charged polyelectrolyte, sodium polystyrene sulfonate (PSS), at pH 5 [71]. The titration curve is presented in Figure 8. The addition of PSS to the suspension of PLAMs–us resulted in a decrease in the EPM values due to formation of the electrostatic complexes and charge compensation. The point with an electroneutral EPM value corresponds to complete surface charge neutralization. The PSS concentration at the neutralization point was found to be (2.96 ± 0.15)∙10^−5^ base-mol/L. From these data, one could calculate the number of available NH_2_ groups on individual micelle as:

(5) $$N_{NH2}=\frac{\nu_{PSS}}{\nu_{PLAMs-us}}=1250\pm 100\;$$

where $\nu_{PSS}$ is molar concentration of monomer units at the neutralization point and $\nu_{PLAMs-us}$ is molar concentration of *PLAMs–us*. 

As was described above, the average number of polylactide molecules in micelles is 6000. So, the fraction of available NH_2_ groups (*X*) could be calculated as:

*Χ* = N_NH2_/(N_AN_ × 0.3) = 0.7 (6)

However, we should take into account the additional surface negative charge that was imparted by the initiator. So, it is more correct to analyze not the point of the electroneutrality but the point with EPM = −0.7 ± 0.1 (μm/s)/(V/cm). Applying the calculations described above, we may estimate that up to 95% of the amino groups are available on the surface of PLAMs–us.

The characteristics of PLAMs–us are summarized in Table 1.

### 3.3. Preparation and Characterization of the Drug-Loaded PLAMs–us

Micelles loaded with antitumor paclitaxel (PTX) were obtained by the addition of the PTX solution in THF to the mixture of the PLA-PEG and PLA-NH_2_ solutions in THF so that the calculated mass fraction of the PTX was 5%. The mixture was vigorously intermixed and the procedure of the preparation of PLAMs–us was applied. As a result, the PLAMs–PTX micelles were prepared. The resulting suspension did not contain precipitate of the water-insoluble PTX, so the added drug was included in micelles [72]. Additional confirmation of the incorporation of PTX in micelles was made by UV–Vis and IR spectroscopy.

**Figure 9:**
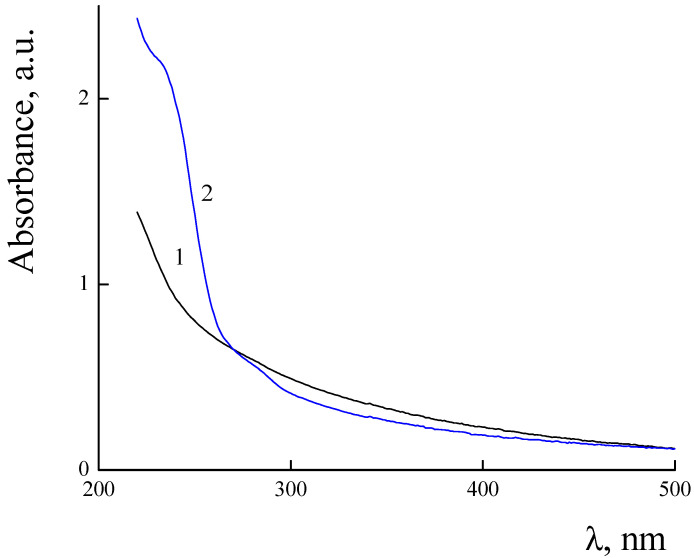
Absorbance spectra of PLAMs–us (1) and PLAMs–PTX (2). Concentration of micelles 0.5 mg/mL, Tris buffer with pH 7, c = 0.01 M, 25 °C.

**Figure 10:**
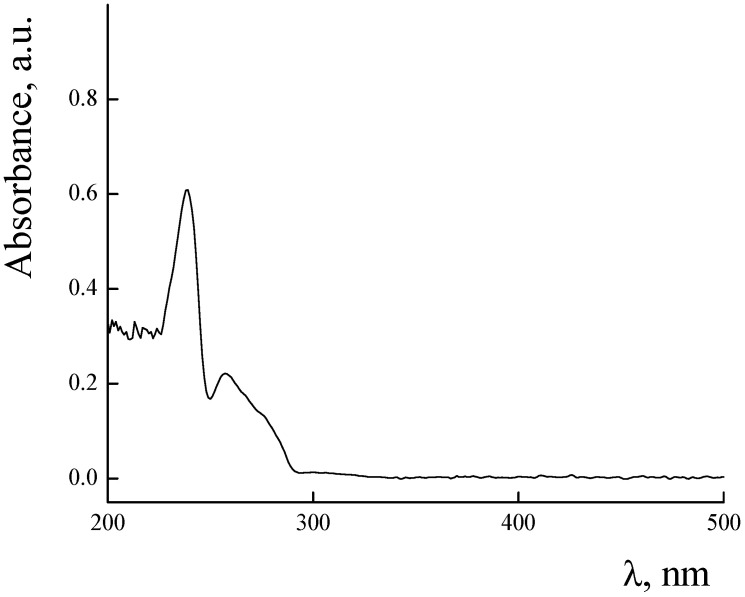
Absorbance spectra of Paclitaxel-Teva. Concentration of Paclitaxel-Teva 0.5 mg/mL, Tris buffer with pH 7, c = 0.01 M, 25 °C.

UV–Vis spectra of PLAMs–us and PLAMs–PTX are presented in Figure 9. The PLAMs–us spectrum does not have any characteristic peaks and the increase in the absorption in the UV region is attributed to overall turbidity of the suspension of micelles (see Figure 9, curve 1). For the PLAMs–PTX sample, the intensive peak at 232 nm was found. According to the literature data for the PTX in THF, the peak at 230 nm corresponds to the absorption of PTX [73]. In Figure 10, the UV–Vis spectrum of commercial Paclitaxel-Teva is presented. As one can see, a sharp peak at 235 nm reflects the presence of PTX in the formulation. 

The IR spectroscopy was used to characterize the PLAMs–PTX system. In addition to peaks corresponding to PLAMs–us at 920–800 cm^−1^ and 1690–1550 cm^−1^, the PLAMs–PTX spectrum contains peaks corresponding to stretching of C=C bonds in the aromatic ring at 1590–1650 cm^−1^ and 1615–1495 cm^−1^ and the peak corresponding to oscillation of Ar-COO-R groups at 1715–1730 cm^−1^ (see Figure S3 in the Supplementary Materials). Thus, the PTX in PLAMs–us micelles is completely solubilized.

**Figure 11:**
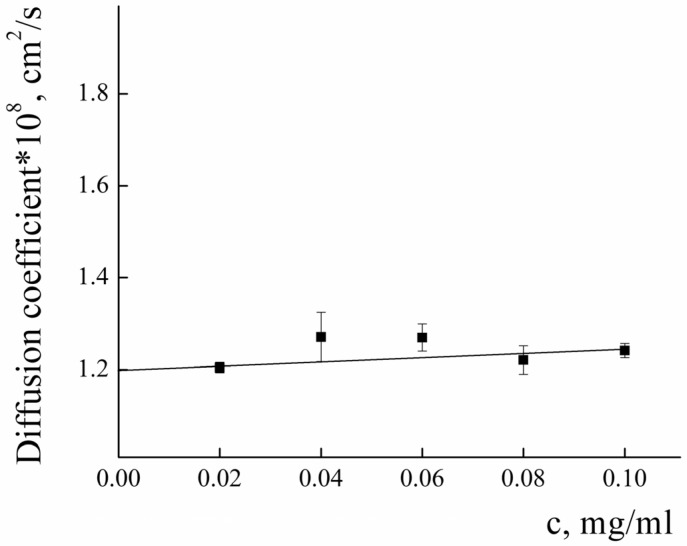
Concentration dependence of the diffusion coefficient of PLAMs–PTX in Tris buffer with pH 7, c = 0.01 M, 25 °C.

The diffusion coefficient and the Rh of the PLAMs–PTX were measured according to the procedure described above (see Figure 11). The value of D_0_ was found to be (1.20 ± 0.01) × 10^−8^ cm^2^/s and the R_h_ value was calculated to be 195 ± 15. The slight decrease in the D_0_ and resulting increase in the R_h_ of PLAMs–PTX in comparison to PLAMs–us could be explained by the increase in the mass of the core of micelles due to incorporation of the PTX.

**Figure 12:**
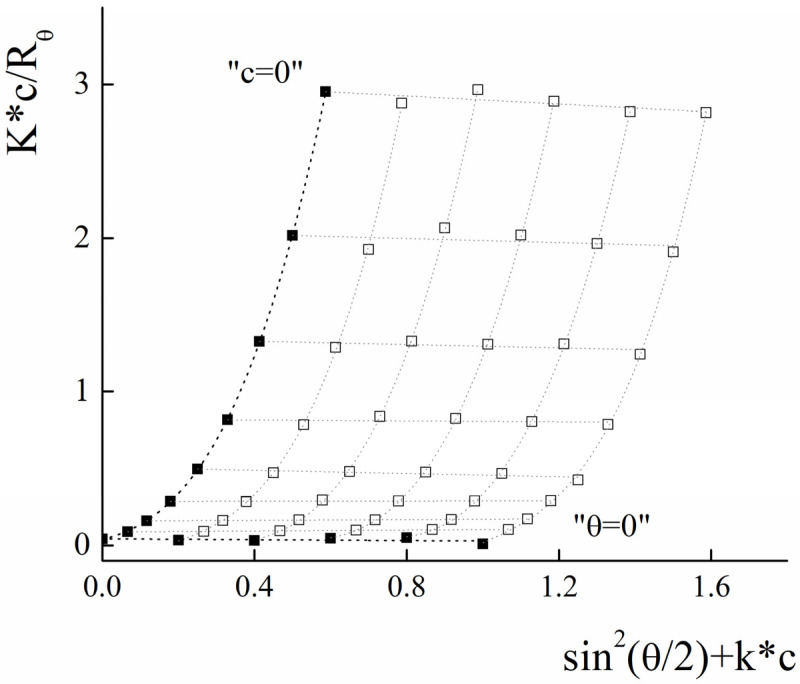
Zimm plot for PLAMs–PTX (blank squares correspond to experimental data, solid squares correspond to extrapolation data). Tris buffer with pH 7, c = 0.01 M, 25 °C.

In accordance with the procedure described above, the radius of gyration and molecular weight were determined for the PLAMs–PTX by analyzing the Zimm plot presented in Figure 12.

The resulting R_g_ was calculated to be 155 ± 20 nm and Mw = (2.30 ± 0.20) × 10^7^ g/mol. The calculated form factor ρ = 0.78 ± 0.15 reflects retaining of the core–shell micelle structure after incorporation of the PTX. Using the formula for calculation of AN and taking into account that 5% of micelle mass is the mass of PTX, we may estimate that the aggregation number for PLAMs–PTX is 6150±550 which is in good agreement with the data obtained for the blank PLAMs–us. 

**Figure 13:**
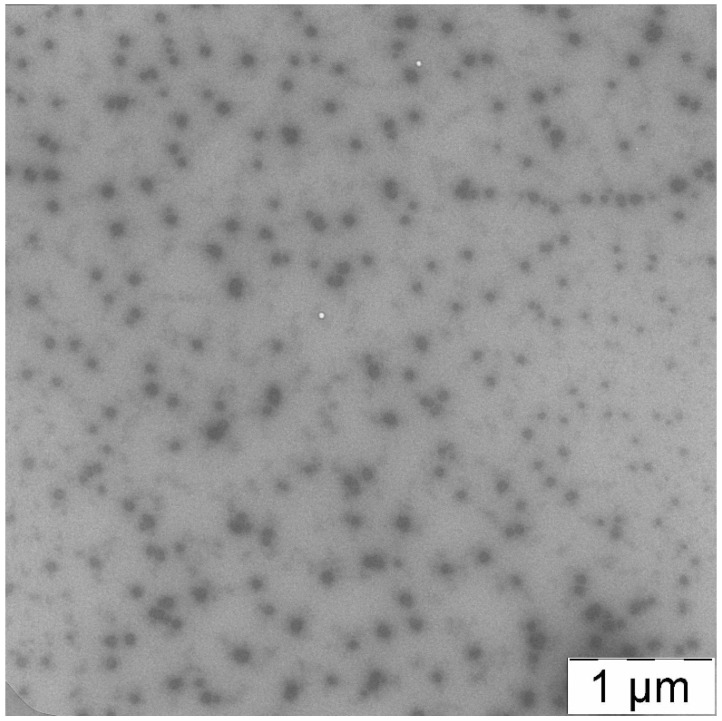
TEM image of PLAMs–PTX.

Retention of the morphology after PTX encapsulation was also confirmed by TEM micrographs (see Figure 13). The “corona” of the hydrophilic block (PEG) and the dense hydrophobic core of PLA are easily observed in the image. So, the structure of micelles represents by itself the “core–shell” type, which is consistent with the obtained values of the shape factor.

Hence, the incorporation of PTX in PLAMs during the procedure of preparation does not affect micelle structure.

The characteristics of PLAMs–us are summarized in Table 2.

### 3.4. Colloid and Enzymatic Stability of the PLAMs

Polylactide-based materials are known to undergo hydrolysis in basic media [74]. Electrolysis and enzymatic hydrolysis have a role in the enhancement of the electrochemical properties of 3D-printed carbon black/poly (lactic acid) structures. The rate of hydrolytic decomposition of a nanocontainer significantly affects the effectiveness of the drug encapsulated in it: the slower the destruction of the container, the higher the therapeutic effect of the biologically active drug. To study the process of violation of the micelle integrity by hydrolysis of polylactide particles in a medium with different pH values, the hydrodynamic sizes of the samples were measured for 1 month. The results can be found in the Figures S4 and S5 in the Supplementary Materials. In slightly acidic media and neutral pH media, no reasonable change in sizes of the micelles was observed during the measurement period. For the samples incubated in media with pH = 9, the average size of the micelles was found to grow in less than 7 days of incubation, reflecting loss of the colloid stability due to disruption of micellar structures.

The samples of PLAMs–PTX were analyzed by measuring their size in suspension incubated in Tris buffer with pH 7. Incorporation of PTX in PLAMs–us did not result in loss of colloid stability for 1 month of observation. 

**Figure 14:**
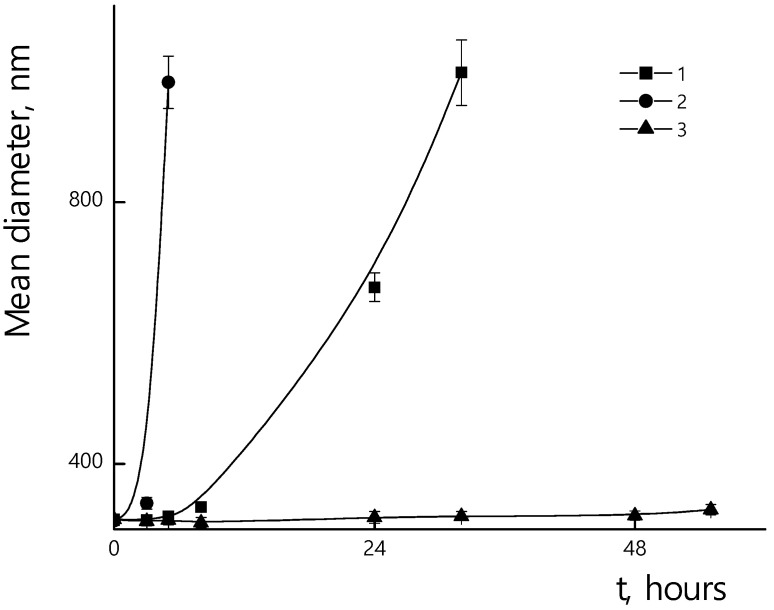
Mean hydrodynamic diameters of PLAMs–us suspensions with trypsin during incubation. Concentration of PLAMs 0.5 mg/mL, concentration of trypsin 4 mg/mL, Tris buffer with pH 7, temperatures 25 °C (1); 37 °C (2); 7 °C (3).

Once introduced into the body, PLAMs are exposed to enzymes. Enzymatic hydrolysis is an unfavorable factor at the initial stage of drug administration, since it reduces the probability of the drug reaching target areas, and also reduces the general therapeutic effect. On the other hand, the biodegradability of nanocontainers is an essential requirement for dosage forms, since after performing their function as a drug carrier, micelles must be effectively removed from the body, and not accumulate in it. Experiments were carried out to evaluate the biodegradability of the nanoparticles. The resistance of polylactide micelles to the enzyme trypsin, which is specific to polylactide, was studied by measuring particle sizes in suspension for several days. The 0.5 mg/mL suspension of PLAMS–us was mixed with trypsin so that the concentration of enzyme was 4 mg/mL. The experiments were carried out in three temperature regimes simulating fridge storage at 7 °C, room temperature storage at 25 °C and human body circulation at 37 °C. A pH of 7 was chosen. The results are shown in Figure 14. From the data obtained, one can conclude that the temperature significantly affects the hydrolysis process. Thus, for individual PLAMs–us after the 24 h of incubation at 25 °C, the size of the initial particles ceased to be recorded and the system contained only aggregates. The formation of large particles could be explained by the fact that during the hydrolysis of the ester bond, a rich mixture of products is formed, some of which (for example, polylactide oligomers) are practically insoluble in water. In this regard, they interact with each other and with surrounding micelles (and other hydrolysis products), which leads to the formation of aggregates [61]. For PLAMs–us incubated at 37 °C, the formation of aggregates occurred 5 h after the start of the experiment. For a temperature regime of 7 °C, no change in the hydrodynamic diameter of PLAMs–us was observed during the entire duration of the experiment.

**Figure 15:**
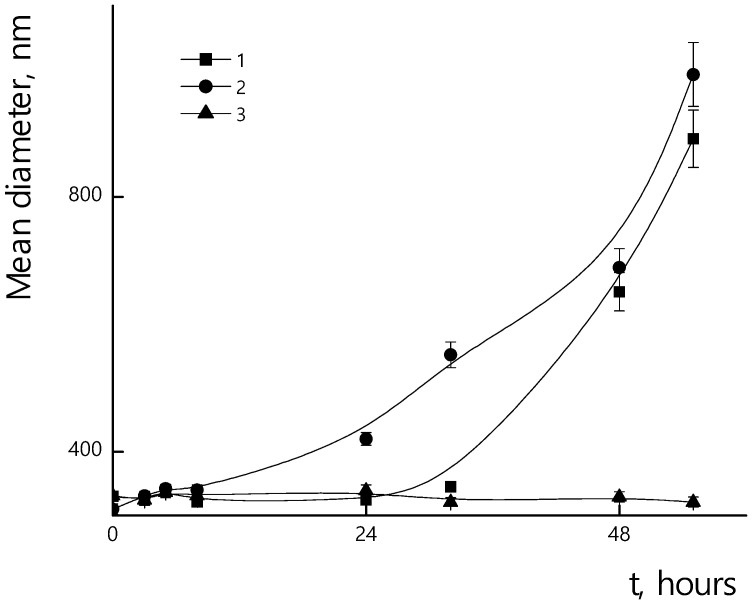
Mean hydrodynamic diameters of PLAMs–PTX suspensions with trypsin during incubation. Concentration of PLAMs 0.5 mg/mL, concentration of trypsin 4 mg/mL, Tris buffer with pH 7, temperatures 25 °C (1); 37 °C (2); 7 °C (3).

Incorporation of the PTX in micelles could affect their resistance towards enzymatic hydrolysis. In Figure 15, the dependences of sizes of PLAMs–PTX/trypsin mixtures over incubation time are presented. A slight increase in stability of PLAMs–PTX was observed at 25 °C and 37 °C in comparison to blank PLAMs–us. So, after 24 h of drug-loaded micelle incubation with the enzyme at 25 °C, samples retained their original size. At 37 °C, a slight increase in particle diameters was observed after 24 h of incubation, and the formation of a large number of large aggregates occurred after 48 h of incubation. At 7 °C, as well as in the case of unloaded PLAMs–us, no change in size was observed for PLAMs–PTX during 56 h of incubation with trypsin. An increase in the time for which PLAMs–PTX particles remain resistant to enzymatic hydrolysis is probably due to the fact that the incorporation of a hydrophobic compound into the micelle core hinders hydrolysis.

### 3.5. Cytotoxicity of the PLAMs

**Figure 16:**
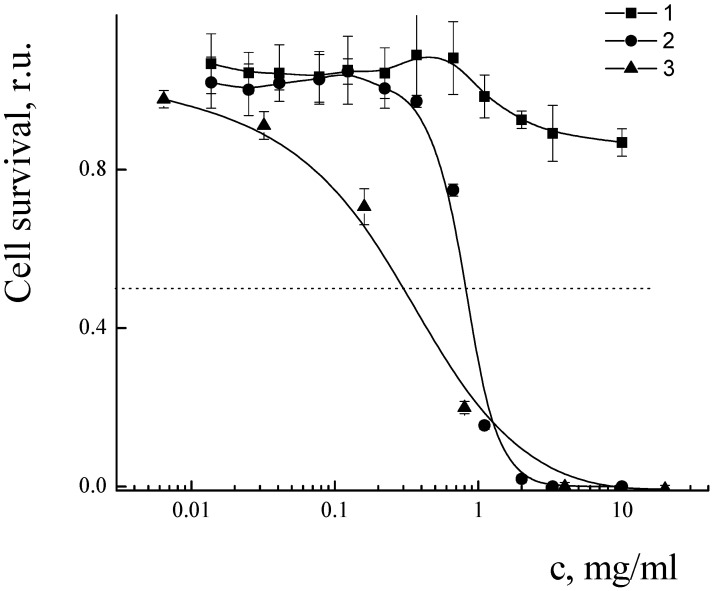
Cell survival after treatment with PLAMs–us (1); PLAMs–PTX (2); and Paclitaxel-Teva (3).

**Figure 17:**
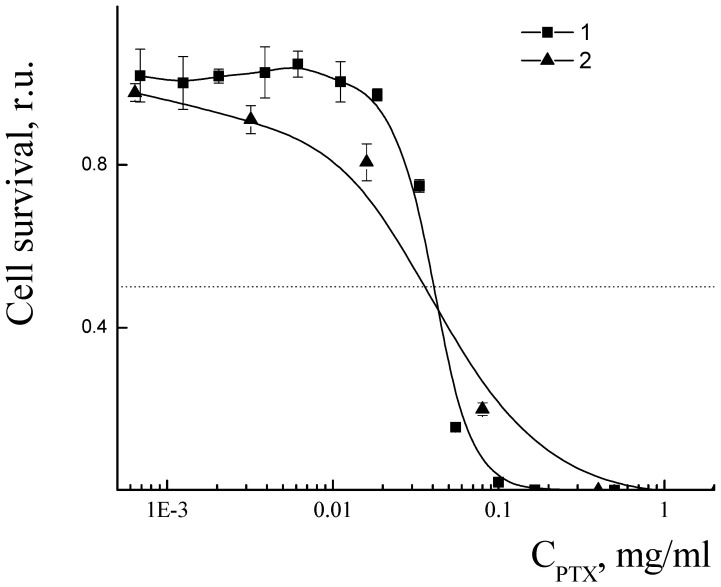
Cell viability in presence of PLAMs–us (1) and Paclitaxel-Teva (2).

The cytotoxicity of PLAMs–us was estimated by an MTT test on the MDR cell line NCI/ADR-RES (formerly designated as MCF-7/ADR) derived from human ovarian cancer Ovcar-8 breast adenocarcinoma cells. The dependence of the relative number of surviving cells upon concentration of micelles is presented in Figure 16. No cytotoxic effect was observed for the concentration of PLAMs up to 10 mg/mL. This result is in good agreement with the cytotoxicity data for the micelles obtained by the standard procedure. Additionally, it should be noted that incorporation of PLA-NH_2_ in micelles did not result in an increase in the cytotoxicity of PLAMs–us as compared to micelles formed from individual PLA-PEG copolymer [75]. So, PLAMs–us have great potential to serve as nanocontainers for substances that carry no cell-killing function. For the drug-loaded PLAMs–PTX, the IC50 reflecting the concentration inducing death of 50% of cells was estimated as 1 mg/mL. The results were compared to the cytotoxicity of the commercial PTX form Paclitaxel-Teva (Figure 16, curve 3). The IC50 for the infusion form of Paclitaxel-Teva was found to be 0.3 mg/mL which is in good agreement with literature data [75,76]. The results of recalculation of MTT test data presented in Figure 16 into the concentration of active substance, PTX, are presented in Figure 17. One can see that the IC50 values of active PTX are almost the same for PLAMs–PTX (IC50 PLAMs–PTX = 42 ± 4 µg/mL) and Paclitaxel-Teva (IC50 Paclitaxel-Teva = 48 ± 7 µg/mL). Thus, PTX encapsulated in mixed polylactide micelles retained the ability to kill cancer cells. Moreover, PTX encapsulated in micellar nanocontainers exhibits a therapeutic effect equal to the commercial drug form. The release of PTX from the PLAMs–PTX during incubation time was measured by UV spectrophotometry. No valuable signal of PTX was detected in solution separated from PLAMs–PTX incubated with PBS buffer either at 5 min or at 1.5 h of incubation.

## 4. Discussion

Mixed polylactide micelles were prepared by a film rehydration technique with application of tip ultrasonication with power 550 W.

The absence of the melting peak for the poly-L-lactide on DSC curves of all samples of PLAMs proves that no local phase separation was observed for the micelles. However, the power of homogenization of the PLAM suspension after film rehydration plays an essential role in preparation of micelles suitable for application as nanocarriers for drug delivery. A slight impact of vortexing or utilization of an ultrasonic bath with low power on the PLAM suspension results in the formation of polydisperse micelles with relatively high mean diameter. The same result was achieved using a standard procedure of solvent substitution. So, the film rehydration method allows, at least, shortening of the time of the polylactide micelle preparation. Application of an ultrasonic tip homogenizer for the dispergation of the rehydrated film of polylactides results in preparation of PLAMs with a narrow size distribution and a mean diameter less than 400 nm. So, such micelles have a great potential for application as antitumor drugs as they are able to accumulate in solid tumors by the EPR effect. The PLAMs–us micelles are spherical core–shell particles with a polylactide core of 70 ± 15 nm and PEG corona. The surface of the PLA core in mixed micelles contains amino groups that are completely charged in acidic media. In neutral media, mixed micelles carry an overall electroneutral charge. Hence, this property could be used for reinforced electrostatic adsorption of PLAMs on the surface of tumor cells as they are surrounded by acidic media [77]. Another possible application of the surface amino groups could be their chemical modification for additional functionalization of micelles. It is important to mention that physicochemical parameters of PLAMs prepared by ultrasonication remain for a long time period. 

Paclitaxel is one of the key drugs in anticancer therapy [78]. Despite a long history of application, there are still problems of side toxicity, low solubility, multidrug resistance and fast clearance of this drug in the human body. So, the search for effective drug delivery forms for PTX is still challenging. The intravenous route of administration of PTX is the major method for treatment [72,73,78,79]. For this purpose, different carriers of amphiphilic and polymeric structures are developing [9,11,15,18,46,61,72,78].

Mixed micelles effectively solubilize hydrophobic biologically active substances [79,80]. The addition of PTX in a mixture of polylactides in organic solvent in the first stage of preparation of PLAMs allows one to easily control the mass fraction of the incorporated drug and its distribution in micelles. Encapsulation of the PTX does not affect size, structure and physicochemical properties of the PLAMs prepared by ultrasonication. The blank and PTX-loaded PLAMs demonstrate good shelf life in neutral and slightly acidic media. Moreover, in the presence of specific enzymes, micelles are able to resist hydrolysis for a reasonable time. At the same time, PLAMs–us and PLAMs–PTX have demonstrated biodegradation. These properties benefit the sufficient circulation time of the PLAMs–based drugs in the human body with consequent decomposition of the carrier after successful delivery of the drug. While blank PLAM nanocarriers demonstrate no cytotoxicity in a wide range of concentrations, the PTX-loaded PLAMs demonstrate the possibility to kill cancer cells with the efficiency of the complex commercial form of PTX.

## 5. Conclusions

For the first time, the advantage of obtaining polylactide micelles by ultrasonic film dispersion was demonstrated and substantiated. 

The stage of evaporation of organic solvent from the mixture of lactides or lactides with PTX supplies uniform intermixing and distribution of the components. The power of sonication plays an essential role in formation of micelles with a small diameter and narrow size distribution. No change in chemical structure of the components during the preparation procedure was detected, so this technique could be applied for the preparation of micelles from different macromolecules. It is important for the compositions containing crystalline and amorphous polymers. Amino-terminated polylactide is arranged in a polylactide core so that most of the amino groups are situated on the surface. At the same time, the net overall charge of the mixed micelles at pH 7 is neutral due to strong screening by the polyethyleneglycol chains. At the same time, in acidic media due to protonation of amino groups micelles obtain positive surface charge that could ensure interaction with negatively charged cell membranes. The size of the micelles, pH sensitivity and low cytotoxicity make them perfect candidates for the creation of drug delivery systems with active and passive targeting possibilities.

The hydrophobic polylactide core of the micelles ensures incorporation of the hydrophobic molecules. Entrapping of the water-insoluble paclitaxel in mixed micelles results in formation of drug delivery nanovehicles with efficiency towards adenocarcinoma cells comparable with the commercially available drug form.

## Figures and Tables

**Table 1:** Major characteristics of PLAMs–us.

Diffusion Coefficient, cm^2^/s	R_h_, nm	Rg, nm	ρ	M_w_	*X*
(1.39 ± 0.01) × 10^−8^	170 ± 15	130 ± 10	0.76 ± 0.12	(2.1 ± 0.1) *×* 10^7^	95%

**Table 2:** Major characteristics of PLAMs–PTX.

Diffusion Coefficient, cm^2^/s	R_h_, nm	Rg, nm	ρ	M_w_
(1.20 ± 0.01) × 10^−8^	195 ± 15	155 ± 20	0.78 ± 0.15	(2.3 ± 0.2) × 10^7^

## Data Availability

Not applicable.

## Supplementary Materials

The following supporting information can be downloaded at: https://www.mdpi.com/article/10.3390/polym14194013/s1, Figure S1. DSC curves for PLAMs–v (1.1); PLAMs–us (2.1); PLAMs–sb (3.1); PLA-PEG (4.1); PLA-NH_2_ (5.1). Heating rate 10 °C/min; Figure S2. DSC curve for PLAMs–cl. Heating rate 10 °C/min; Figure S3. IR spectra of PLAMs–us and PLAMs–PTX; Figure S4. Time dependence of hydrodynamic diameters of PLAMs–us in buffers with pH = 5 (blue), pH = 7 (red) and pH = 9 (yellow). PLAMs–us concentration 0.5 mg/mL; Figure S5. Time dependence of hydrodynamic diameters of PLAMs–us and PLAMs–PTX in buffer with pH = 7 at 7 days (blue), 14 days (red), 21 days (gray) and 30 days (yellow). PLAMs–us and PLAMs–PTX concentrations 0.5 mg/mL.
