# Supplementary material for: Ultrasonic Film Rehydration Synthesis of Mixed Polylactide Micelles for Enzyme-Resistant Drug Delivery Nanovehicles
Source: Polymers (Basel). 2022 Sep 25;14(19):4013. doi: 10.3390/polym14194013 (PMC9571646; doi:10.3390/polym14194013)
Supplement: Supplementary file 1 [file polymers-14-04013-s001.zip › polymers-1912978-supplementary.pdf]

Supplementary Materials

for

Ultrasonic Film Rehydration Synthesis of Mixed Polylactide Micelles for Enzyme-Resistant Drug Delivery Nanovehicles

Darya A. Stepanova [1], Vladislava A. Pigareva [1], Anna K. Berkovich [1], Anastasia V. Bolshakova [1,2], Vasiliy V. Spiridonov [1], Irina D. Grozdova [1] and Andrey V. Sybachin [1,*]

Differential scanning calorimetry measurements were performed using Netzsch DSC 204 F1 (Germany) at heating rate 10 °C/min. The samples of PLAMs were lyophilized prior measurements and 2 mg of samples were used. For the PLA-NH2 and PLA-PEG samples no additional manipulations prior DSC measurements were made. The results were analyzed using software supplied by manufacturer.

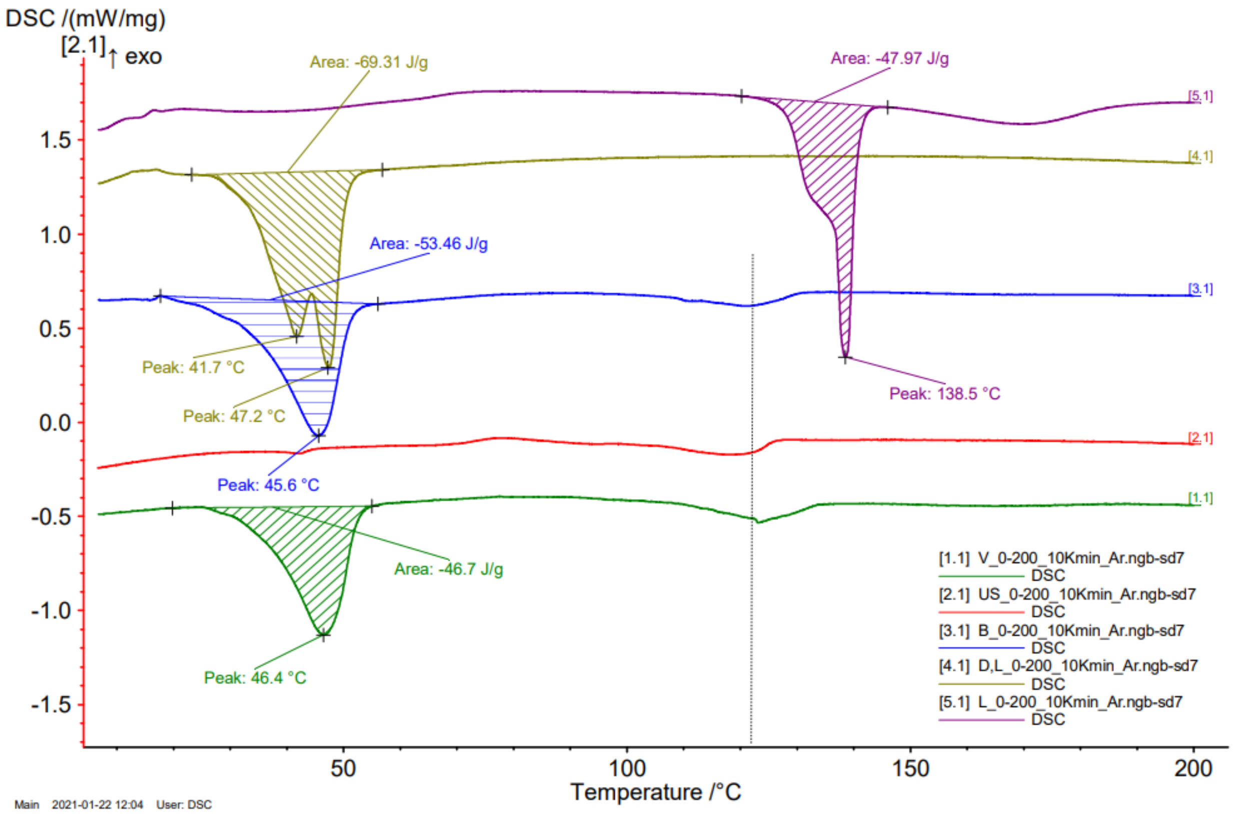

Figure S1. DSC curves for PLAMs-v (1.1); PLAMs-us (2.1); PLAMs-sb (3.1); PLA-PEG (4.1); PLA-NH2 (5.1). Heating rate 10 °C/min.

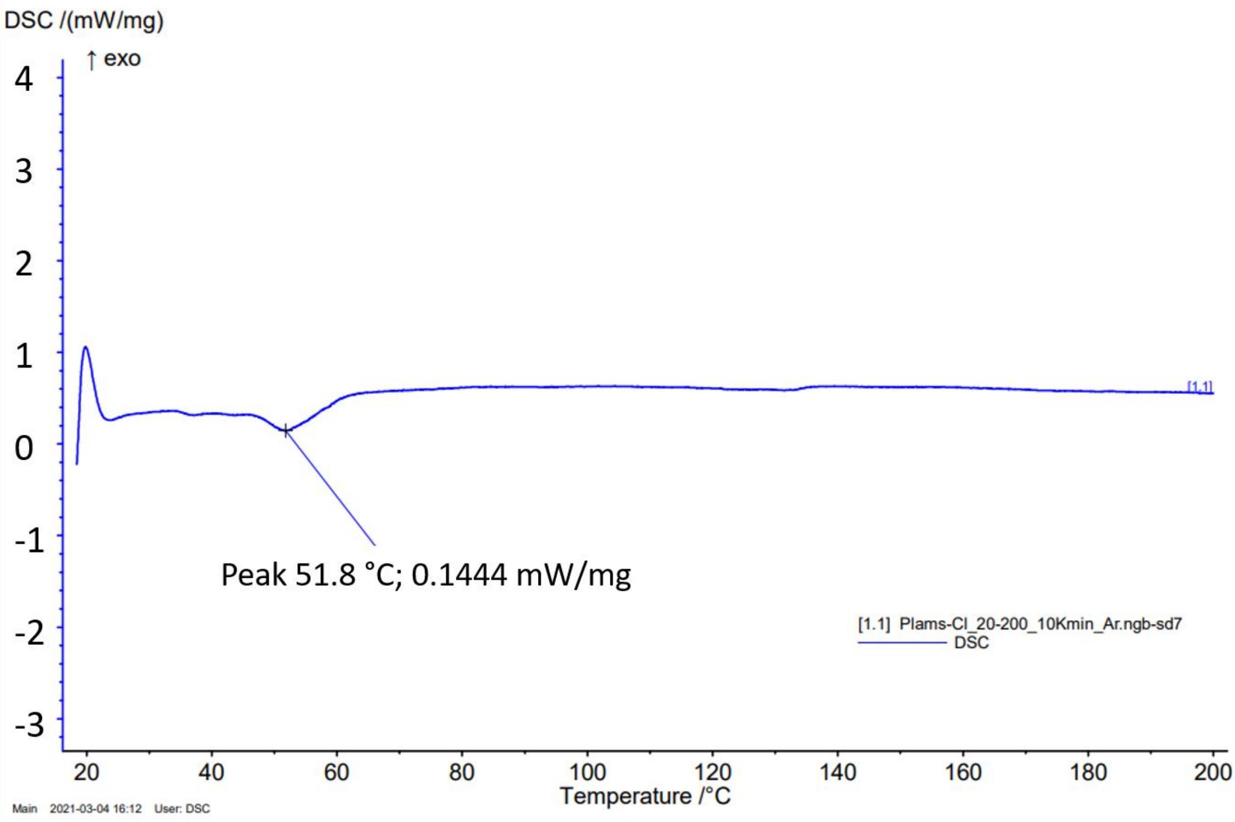

Figure S2. DSC curve for PLAMs-cl. Heating rate 10 °C/min.

IR-spectra of samples were obtained using Carl Zeiss Jena Specord M-80 IR (Germany). The 20 µl droplet of PLAMs suspension with concentration 1 mg/ml was deposited between two KBr glasses and the spectrum was recorded in absorption regime in wavelength range from 400 to 2000 cm^{-1}.

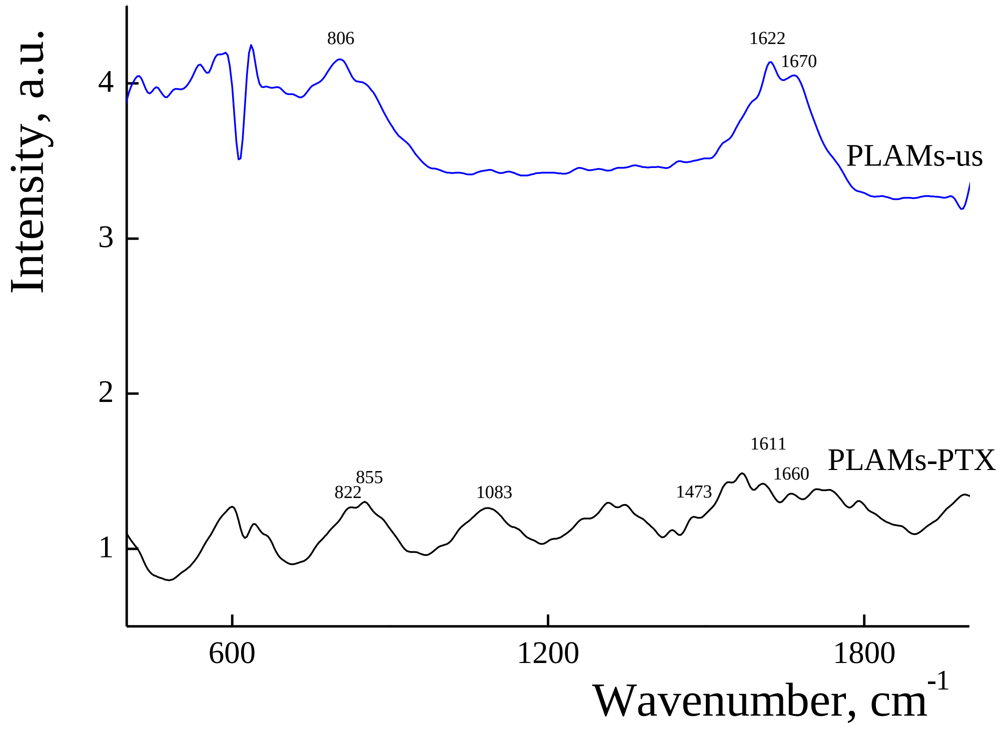

Figure S3. IR-spectra of PLAMs-us and PLAMs-PTX.

The hydrodynamic diameters of PLAMs were obtained by dynamic ligh-scattering using Brookhaven ZetaPlus (Brookhaven, USA) equipment and DynaLS software pakage for size distribution analysis

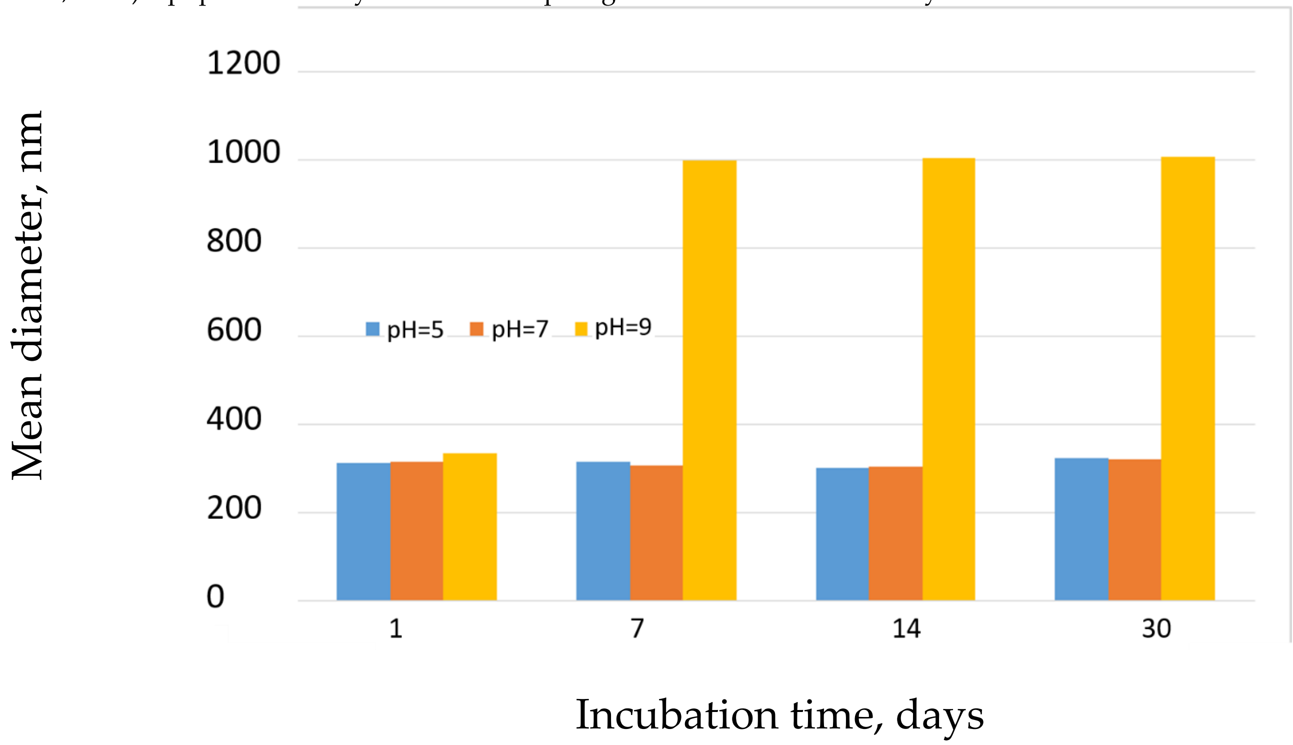

Figure S4. Time dependence of hydrodynamic diameters of PLAMs-us in buffers with pH = 5 (blue), pH = 7 (red) and pH = 9 (yellow). PLAMs-us concentration 0.5 mg/ml.

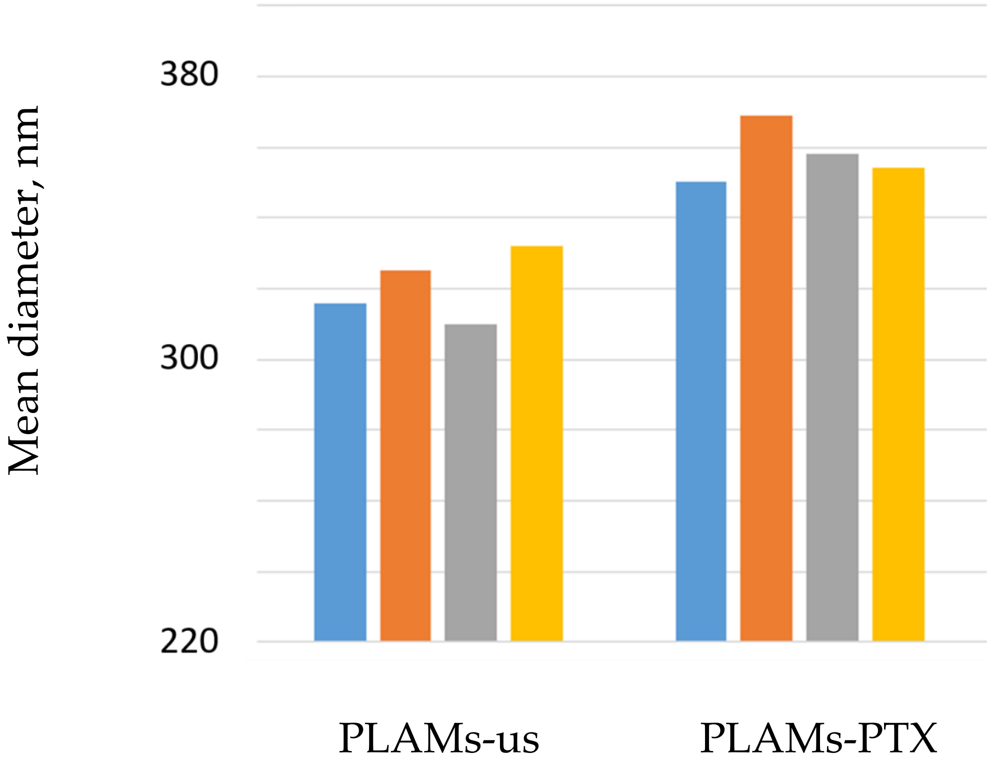

Figure S5. Time dependence of hydrodynamic diameters of PLAMs-us and PLAMs-PTX in buffer with pH = 7 at 7 days (blue), 14 days (red) , 21 day (grey) 30 days (yellow). PLAMs-us and PLAMs-PTX concentrations 0.5 mg/ml.
